# Enhanced bacteriostatic effects of phage vB_C4 and cell wall-targeting antibiotic combinations against drug-resistant *Aeromonas veronii*

**DOI:** 10.1128/spectrum.01908-24

**Published:** 2025-01-16

**Authors:** Xin Cao, Yanqiong Tang, ZhenZhang Lu, Xiang Ma, Hong Li, Xue Chi, Juanjuan Li, Zhu Liu

**Affiliations:** 1 School of Life and Health Sciences, Hainan University 74629 , Haikou, China; 2 Yunnan Provincial Key Laboratory of Animal Nutrition and Feed, Faculty of Animal Science and Technology, Yunnan Agricultural University, Kunming, China; South China Sea Institute of Oceanology Chinese Academy of Sciences, Guangzhou, Guangdong, China

**Keywords:** bacteriostatic effect, phage therapy, bioinformatics, antibiotics, biofilm elimination

## Abstract

*Aeromonas veronii* is a vital zoonotic pathogen known for its extensive drug resistance and ability to form biofilms, which contribute to its antibiotic resistance. In this study, the phage vB_C4, specifically targeting *A. veronii*, was isolated and subjected to bioinformatic analysis and bacteriostatic activity assays. The combination of phage vB_C4 with antibiotics such as cephalothin and cefoxitin, which target the bacterial cell wall, resulted in a significantly enhanced bacteriostatic effect compared to either the phage or antibiotics alone. Furthermore, the phage dosage was critical in optimizing the antimicrobial effect when used in conjunction with antibiotics. This combined treatment exhibited a more distinct effect in removing mature biofilms and inhibiting biofilm formation, leading to a considerable decrease in bacterial density within the biofilm. Overall, the synergistic use of phage and antibiotics offers a novel attitude for treating pathogenic bacteria and holds significant potential in preventing the emergence of drug-resistant strains.

**IMPORTANCE:**

The combined application of phages and antibiotics not only effectively inhibits the emergence of phage-resistant bacteria but also reduces the required effective concentration of antibiotics. Additionally, this combination therapy demonstrates significant therapeutic effects on clinical infections mediated by biofilms. Consequently, this study establishes a basis for evaluating the parameters essential for utilizing phage-antibiotic combination therapy in the treatment of biofilm-associated infections, thereby offering a novel selection for the clinical management of multidrug-resistant bacterial infections.

## INTRODUCTION

*Aeromonas veronii* is a conditionally pathogenic bacterium that infects humans and other mammals, leading to gastroenteritis, septicemia, and traumatic infections, thus posing a significant threat to human health. Immunocompromised individuals, such as those with AIDS, malignant tumors, liver cirrhosis, and diabetes mellitus, are at a heightened risk of mortality upon infection (1). In the past, antibiotics have been the effective method for preventing and treating bacterial infectious diseases. However, the widespread misuse of antibiotics has led to growing drug resistance among pathogenic bacteria, which has become a global health issue (2). *A. veronii* exhibits multidrug resistance when treated with antibiotics, complicating prevention and treatment efforts. This challenge has spurred researchers to explore new therapeutic options for pathogenic bacterial infections (3). Phage-antibiotic combination therapy enhances the effectiveness of antibiotic treatment, reduces the required dosage and concentration of antibiotics, and prevents the emergence of drug-resistant bacteria (4).

Phages (bacteriophages) are viruses that infect microorganisms, including bacteria and fungi. Phage therapy was employed to treat bacterial infections as early as 20 years before the discovery of antibiotics (5). In recent years, the irrational use of antibiotics has exacerbated issues of bacterial multidrug resistance and drug residues, drawing renewed attention to phage therapy (6). Compared to antibiotic treatment alone, phage therapy offers advantages such as strong host specificity, high biosafety, effective bactericidal action, and minimal environmental impact (7). Phages have garnered particular interest as alternatives to antibiotics in areas such as food safety, agriculture, and the agro-environment (8).

Regrettably, bacteria can develop resistance to phages during phage therapy (9). However, the combination of phages and antibiotics not only reduces the concentration of antibiotics required but also inhibits the emergence of phage-resistant bacteria. This combination presents a promising solution to the current challenges in phage therapy (10, 11). Due to the cumulative effect or phage-antibiotic synergy (PAS), the combined use of phages and antibiotics is more effective than either treatment alone in controlling pathogenic bacteria and enhancing bacterial clearance (12–14).

The formation of bacterial biofilms is considered one of the primary reasons for the development of antibiotic resistance in bacteria (15). A biofilm is defined as a community of microorganisms that are associated with surfaces or adhered to each other, living within an extracellular polysaccharide matrix. Biofilms impede drug penetration through their matrix and reduce the ability of antibiotics to reach the bacterial cell surface. Additionally, biofilms enable bacteria to evade the host immune system (16). Phages encode specific enzymes, such as endolysins, Virion-associated peptidoglycan hydrolases, and depolymerizing enzymes, which are effective against biofilms (17). For instance, phage depolymerase degrade polysaccharides in biofilms, including capsular polysaccharides, lipopolysaccharides, and extracellular polysaccharides, as well as degrading peptides or lipids (18). This enables phages to penetrate the biofilm and disrupt its structure, allowing antibiotics to reach deep into the biofilm and effectively eliminate pathogens (19, 20). Numerous studies have shown that the combined use of phages and antibiotics significantly reduces the density of pathogenic bacteria within biofilms and limits the evolution of resistance to both antibiotics and phages (21). For example, the combination of phages and ciprofloxacin effectively eradicates *Klebsiella pneumoniae* within biofilms and prevents the emergence of drug-resistant mutants (22). Similarly, the combination of phages and vancomycin leads to an 87% reduction in biofilm volume, with vancomycin-resistant *Enterococcus* reduced to undetectable levels (23).

*A. veronii* is prone to biofilm formation and exhibits a broad spectrum of drug resistance. Consequently, finding effective methods to eliminate this pathogen is an important and pressing issue. In our study, we performed genome sequencing and bioinformatics analysis of a strain of phage vB_C4 that specifically targets *A. veronii*, with a focus on enzymes that degrade extracellular polymeric substances (EPSs), such as endolysins and hydrolases. The effects of phage vB_C4 in combination with cephalothin or cefoxitin on the removal of mature biofilms and inhibition of biofilm formation were evaluated through *in vitro* antimicrobial activity assays. This research provides novel insights for the treatment of drug-resistant pathogens, particularly *A. veronii* infections.

## RESULTS

### Genomic analysis of the phage vB_C4

**Fig 1:**
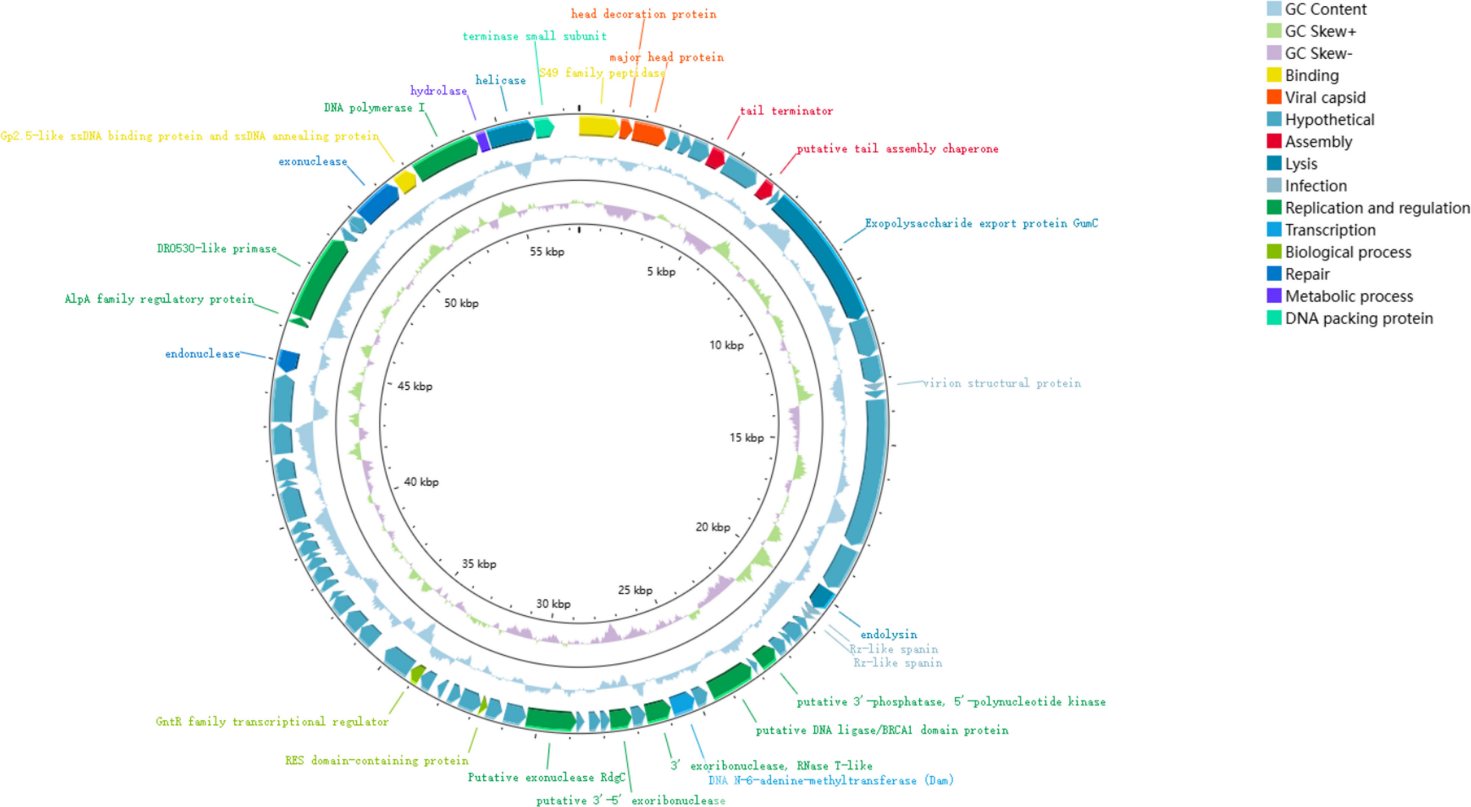
Genome map of vB_C4.

The complete genome of phage vB_C4 was sequenced using the Illumina NovaSeq 6000 platform and subsequently assembled and corrected to obtain its complete genome (Fig. 1). The vB_C4 genome has a size of 60,737 bp with a GC content of 61.39% and does not contain non-coding RNA (ncRNA). A total of 77 coding genes were predicted, including 31 proteins with known functions and 46 annotated as hypothetical proteins. The proteins with known functions are involved in various physiological activities of the phage, such as capsid proteins, tail proteins, structural proteins, DNA replication and regulation, packaging proteins, and proteins related to metabolism and assembly. No genes associated with potential therapeutic limitations, such as antibiotic resistance genes, toxin-encoding genes, or integrases, were identified, suggesting that vB_C4 holds potential for clinical therapeutic applications. Notably, bacterial lysis-related proteins were identified in the phage vB_C4 genome, including GumC, a protein involved in cell wall lysis and a hydrolase that degrades bacterial surface polysaccharides and extracellular polymeric substances (EPSs), and phage lysin-endolysin, which are effective against biofilms and enhance the penetration of antibiotics into biofilms.

### Comparative genomic analysis of phage vB_C4

**Fig 2:**
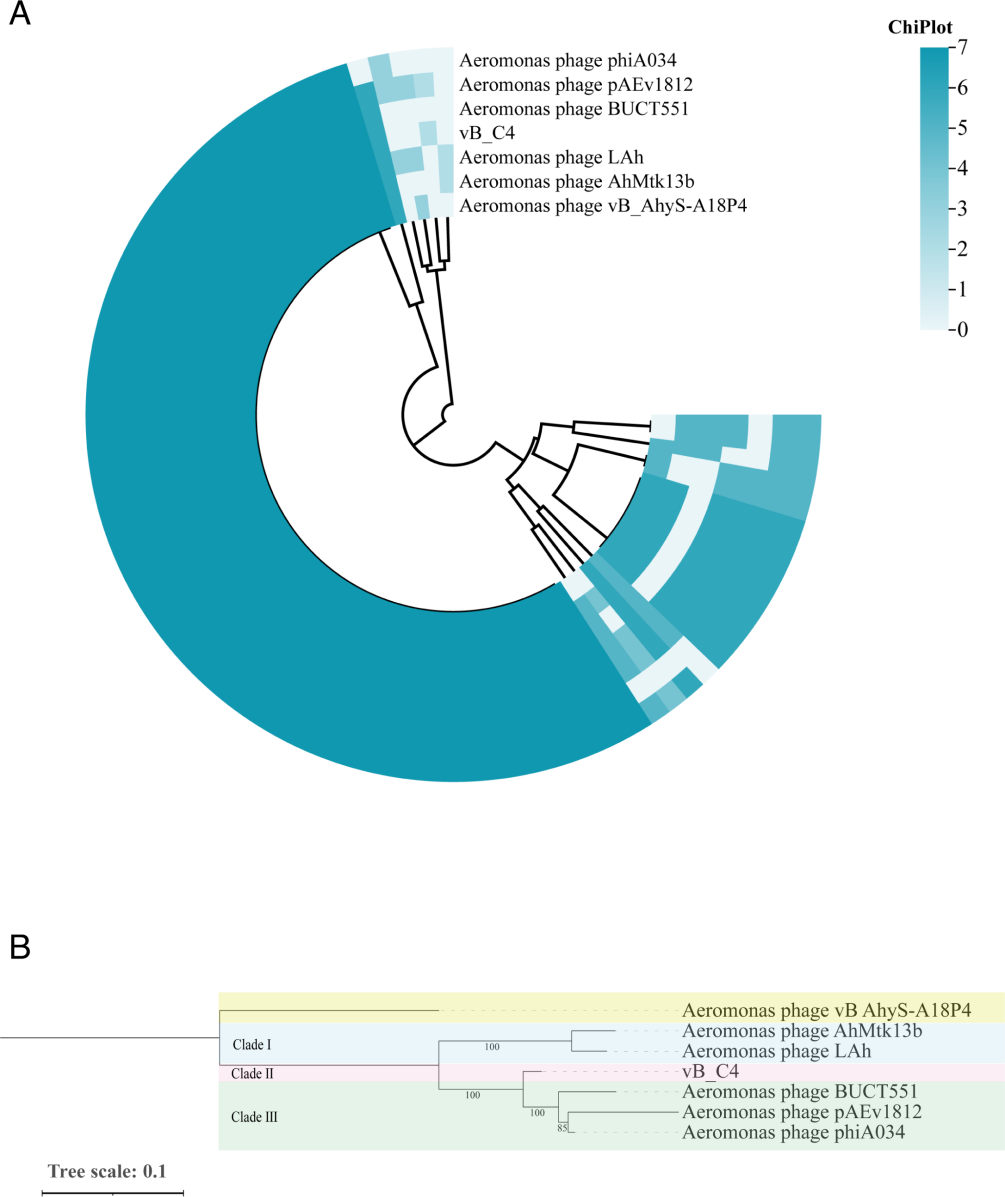
Genomic analysis of phage vB_C4. (**A**) Circular heatmap representing the number of homologous genes among seven phages. The color intensity indicates the number of homologous genes in each phage. (**B**) Phylogenetic analysis of phage vB_C4. The phylogenetic tree was constructed using the maximum likelihood (ML) method with 1,000 bootstrap replicates. The position of phage vB_C4 is indicated by a red box.

Genome-wide comparisons using BLASTN revealed that vB_C4 shares high nucleotide identity (>95%) with other *Aeromonas* phages, including *Aeromonas* phage phiA034 (OP792756.2), *Aeromonas* phage BUCT551 (NC_052986.1), and *Aeromonas* phage pAEv1812 (OL964754.1). Six phage strains with high nucleotide identity to vB_C4 were selected for comparative genomic analysis. A total of 529 homologous genes were identified, comprising 58 single-copy core genes, 18 auxiliary genes, and no specific genes (Fig. 2A). The core genes encoded proteins associated with the phage shell structure, such as head-tail adaptor proteins, head proteins, head decoration proteins, and tail terminator proteins. Additionally, they included enzymes related to basic phage physiological functions, such as helicase, exonuclease, DNA ligase, DNA polymerase, and terminating enzymes.

The phylogenetic tree of seven phages was constructed using the maximum likelihood method (Fig. 2B), revealing three distinct clades. Phage vB_C4 was located in the second clade, while BUCT551, pAEv1812, and phiA034 were located in the third clade, with BUCT551 representing the ancestral branch of pAEv1812 and phiA034. Phages in Clades II and III were found to have a close evolutionary distance, sharing a recent common ancestor. AhMtk13b and LAh were positioned in the first clade, indicating an earlier divergence and a closer evolutionary relationship compared to the other two clades.

### *In vitro* safety assessment of phage vB_C4 and its ability to inhibit *A. veronii* C4

**Fig 3:**
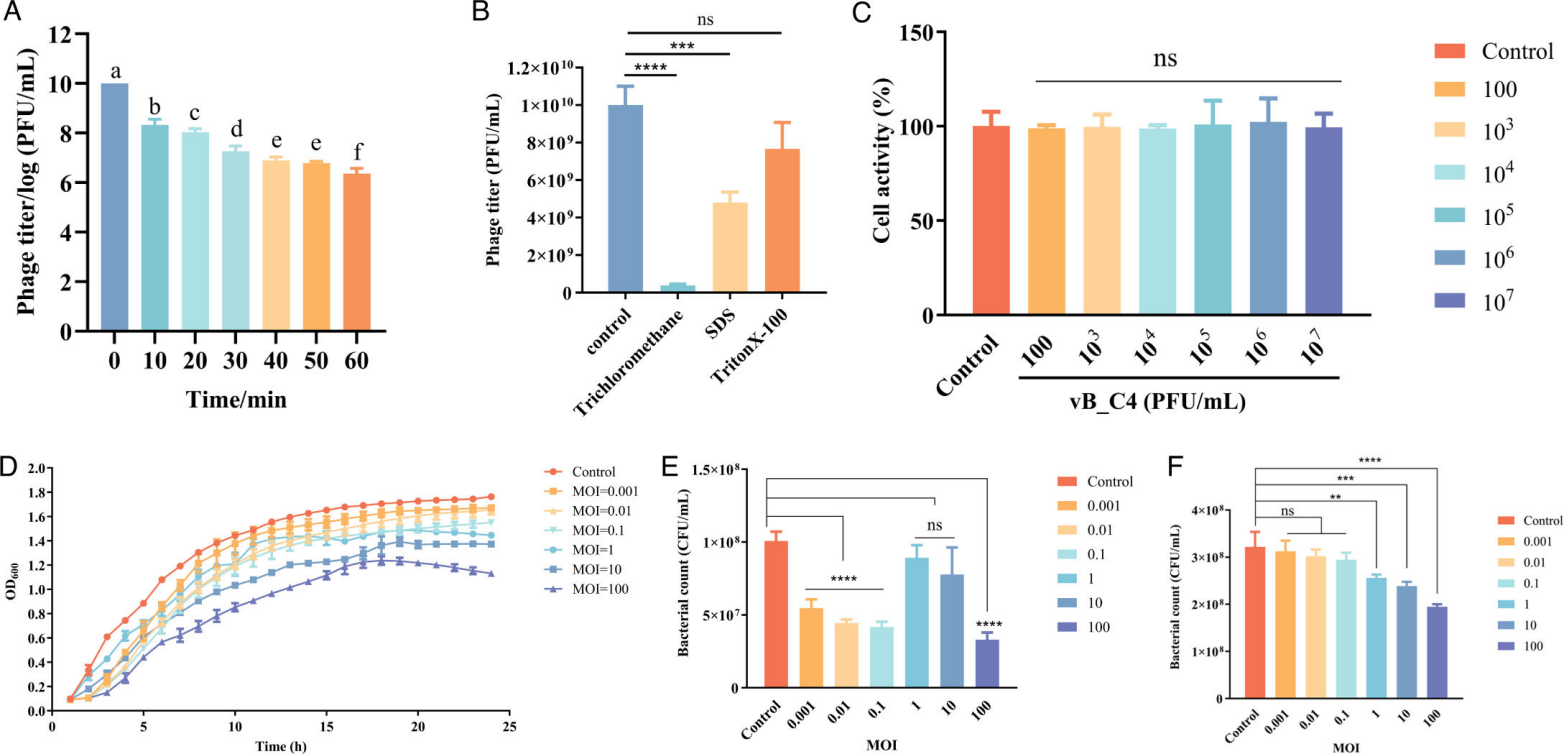
Biological characteristics of phage vB_C4. (**A**) The effect of UV light on phage activity. (**B**) The effect of organic solvents on phage activity. (**C**) Cytotoxicity of phage vB_C4 on intestinal epithelial Caco-2 cells. (**D**) Bacteriostatic effect of phage vB_C4 against *A. veronii* C4 at different growth stages *in vitro*. (**E**) Bacteriostatic results of phage vB_C4 at 3 h *in vitro*. (**F**) Bacteriostatic results of phage vB_C4 at 24 h *in vitro*.

We first determined the bacterial lysis spectrum of phage vB-C4. As shown in Table 1, phage vB-C4 exhibits lytic activity toward *A. veronii*, but not toward common pathogens such as *Staphylococcus aureus*, *Salmonella* S504, *Streptococcus agalactiae*, *Vibrio alginolyticus*, and *Pseudomonas aeruginosa*. The results indicate that phage vB-C4 demonstrates good specificity. In addition, vB_C4 exhibited reduced lytic activity after treatment with chloroform and the descaling agent SDS, but it was tolerant to the descaling agent TritonX-100 and UV light (Fig. 3A and B).

**TABLE 1:** Host range of phage vB_C4^*a*^

Strains	Results
*Aeromonas veronii*	+
*Staphylococcus aureus*	−
*Salmonella* S504	−
*Streptococcus agalactiae*	−
*Vibrio alginolyticus*	−
*Pseudomonas aeruginosa*	−

^*a*^ “+”，Cracking；“−”， not cracking.

The cytotoxicity of phage vB_C4 on intestinal epithelial Caco-2 cells was initially assessed, as depicted in Fig. 3C. Caco-2 cells were treated with varying doses of phage vB_C4 for 24 h. No significant difference was observed between the treatment and control groups, indicating that phage vB_C4 did not exhibit notable toxicity toward animal cells.

To evaluate the antibacterial efficacy of vB_C4 against *A. veronii* C4, the host bacteria were co-cultured with different doses of the phage *in vitro* (Fig. 3D). The negative control continued to grow, reaching a maximum optical density at 600 nm (OD_600_) value of 1.8 at 24 h. At low phage concentrations (multiplicity of infection [MOI] ≤ 0.1), the phage exhibited some inhibitory effect early in bacterial cultivation, but this effect diminished with prolonged culture time. At medium phage concentrations (MOI between 1 and 10), no significant antibacterial effect was observed at the early stages; however, the growth rate of *A. veronii* C4 was significantly inhibited as the culture time was extended. At high phage concentrations (MOI of 100), bacterial growth was inhibited from the early stages and the antibacterial effect persisted throughout the entire culture period (Fig. 3D and E). After 24 h of co-cultivation, both medium and high doses (MOI ≥ 1) of phage significantly reduced the colony count of *A. veronii* C4, whereas lower doses (MOI ≤ 0.1) were ineffective in inhibiting the growth and reproduction of *A. veronii* C4 (Fig. 3F).

### The effect of combined use of antibiotics and phage on planktonic bacteria of *A. veronii* C4

**Fig 4:**
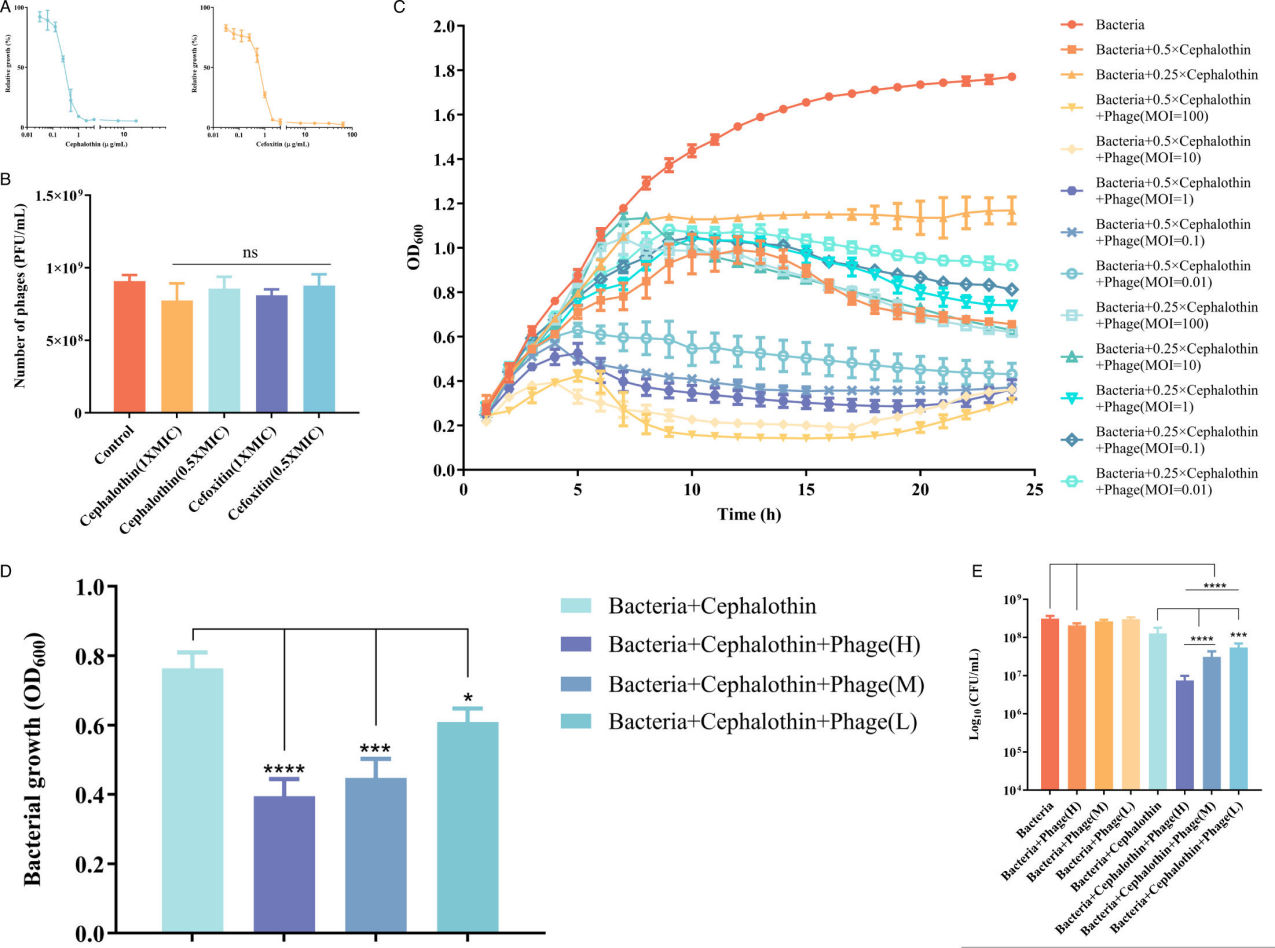
Effect of combination of phage vB_C4 and cell wall-targeting antibiotics on *A. veronii* C4. (**A**) The minimum inhibitory concentration (MIC) of antibiotics against *A. veronii* C4. (**B**) The effect of antibiotics on the proliferation of phage vB_C4. (**C**) The effect of cephalothin combined with phage on the growth of *A. veronii* C4. (**D**) Bacterial turbidity (OD_600_) at 6 h of treatment with cephalothin combined with phage. (**E**) Plate counts at 20 h of treatment with cephalothin in combination with phage. L indicates low dose (MOI = 0.01); M indicates medium dose (MOI = 1); and H indicates high dose (MOI = 100). Data are represented as the mean of three biological replicates, with error bars representing the standard deviation (SD) of the mean. * indicates *P* < 0.05, ** indicates *P* < 0.01, *** indicates *P* < 0.001, **** indicates *P* < 0.0001, and NS indicates no significant difference.

To evaluate the antibacterial activity against *A. veronii* C4, antibiotics cephalothin and cefoxitin, which target the cell wall, were combined with phage vB_C4. As depicted in Fig. 4A, the minimum inhibitory concentration (MIC) of cephalothin and cefoxitin against *A. veronii* C4 was 1 and 2 µg/mL, respectively. Based on these MIC values, we assessed whether the antibiotics themselves affected phage activity. According to the phage counts (Fig. 4B), the number of phage plaques remained approximately the same before and after the addition of antibiotics, with no statistically significant difference (*P* > 0.05). Therefore, the selected antibiotic concentrations did not affect the potency of the phage.

**Fig 5:**
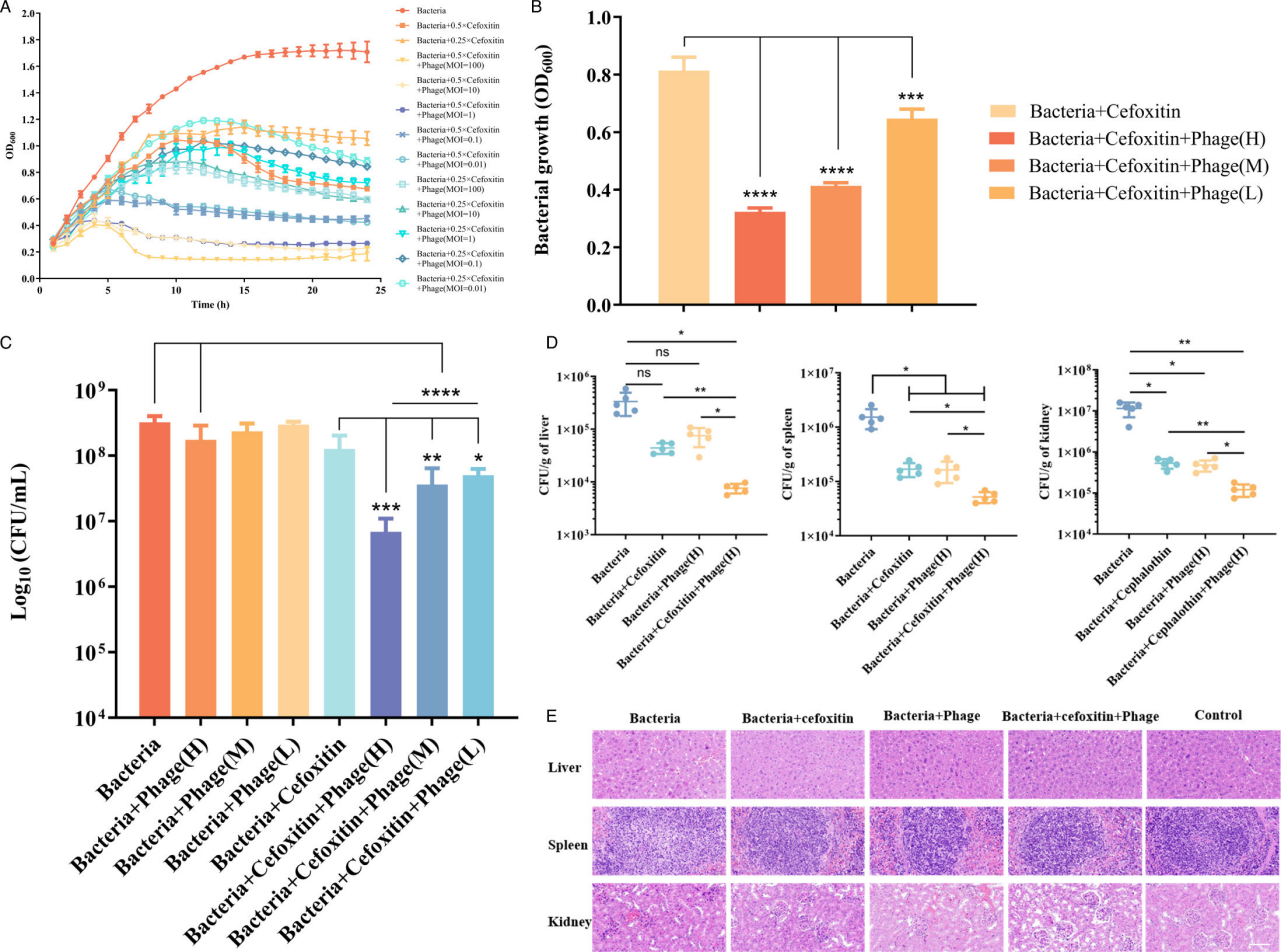
The effect of the combination of phage vB-C4 and cefoxitin on *A. veronii* C4. (**A**) The effect of cefoxitin combined with phage on the growth of *A. veronii* C4. (**B**) Bacterial turbidity (OD_600_) at 6 h of treatment with cefoxitin combined with phage. (**C**) Plate counts at 20 h of treatment with cefoxitin in combination with phage. (**D**) Count of bacteria in the organs of mouse tissues. The data are displayed as mean ± standard deviation, ns indicates no significant difference, *n* = 5. (**E**) Histological examination (H&E) of pathological changes in mouse tissues and organs. The scale bar displays the indicated length (50 µm). L indicates low dose (MOI = 0.01); M indicates medium dose (MOI = 1); and H indicates high dose (MOI = 100). Data are represented as the mean of three biological replicates, with error bars representing the standard deviation (SD) of the mean. * indicates *P* < 0.05, ** indicates *P* < 0.01, *** indicates *P* < 0.001, **** indicates *P* < 0.0001, and NS indicates no significant difference.

When cephalothin is used in combination with phage vB-C4, the combination of subinhibitory concentration of cephalothin (0.5 × MIC) and phage combination have the most significant effect (Fig. 4C), and similar results were obtained with cefoxitin (Fig. 5A). Therefore, subsequent experiments will focus on the antibacterial effect of different doses of phage vB_C4 combined with subinhibitory concentrations of antibiotics and the effect on biofilm formation. When cephalothin was used in combination with different doses of phage vB_C4, a significant antibacterial effect was observed with low-dose phage (MOI = 0.01), and the antibacterial activity was even more pronounced with medium-dose (MOI = 1) and high-dose (MOI = 100) phages at 6 h (Fig. 4C and D). A similar trend was observed with the combination of cefoxitin and phage vB_C4 at 6 h(Fig. 5A and B). Similar results were obtained by counting the dilution plates of bacteria treated with bacteriophages and antibiotics for 20 h. When phage vB_C4 was applied in combination with sub-inhibitory concentrations of cephalothin, the low-dose (MOI = 0.01), medium-dose (MOI = 1), and high-dose (MOI = 100) combination groups significantly inhibited the growth of *A. veronii* C4 compared to the control, antibiotics alone, or high-dose phage alone, resulting in a significant synergistic effect (Fig. 4E). Similarly, when phage vB_C4 was applied in combination with sub-inhibitory concentrations of cefoxitin, there was a significant decrease in bacterial growth in the co-application group compared to the control, as well as a significant difference compared to antibiotic or high-dose phage treatment alone (Fig. 5C).

In order to assess the therapeutic efficacy of phage vB_C4 in combination with antibiotics against *A. veronii* infections, a high dose (MOI = 100) of phage in combination with sub-inhibitory concentration of cefoxitin was administered to the infected group *in vivo* to treat the infection in mice. The results indicated that the combination treatment exerted a significant bactericidal effect on *A. veronii* C4 in the liver, spleen, and kidney of mice (Fig. 5D). In addition, compared with the infected group, the treatment groups with high dose (MOI = 100) of phage vB_C4 alone or cefoxitin alone demonstrated significant inhibitory effects on the growth of *A. veronii* C4 only in the spleen and kidney (Fig. 5D). Histopathological analysis revealed that infection with *A. veronii* resulted in the damaged and ablated nuclei of the liver, accompanied by hemorrhage. The number of lymphocytes in the white pulp of spleen tissue decreases, exhibiting punctate lymphocyte necrosis and nuclear fragmentation. The glomeruli in the kidney area are enlarged, indicating kidney damage in mice. Following treatment, the morphology of liver cells appeared normal, and there was no significant change in the number and size of white pulp lymphocytes in spleen tissue. The kidney structure was intact, and the glomerular morphology was well-defined (Fig. 5E).

### Effect of the combination of vB_C4 and antibiotics *in vitro* activity against both mature biofilm and biofilm formation of *A. veronii* C4

**Fig 6:**
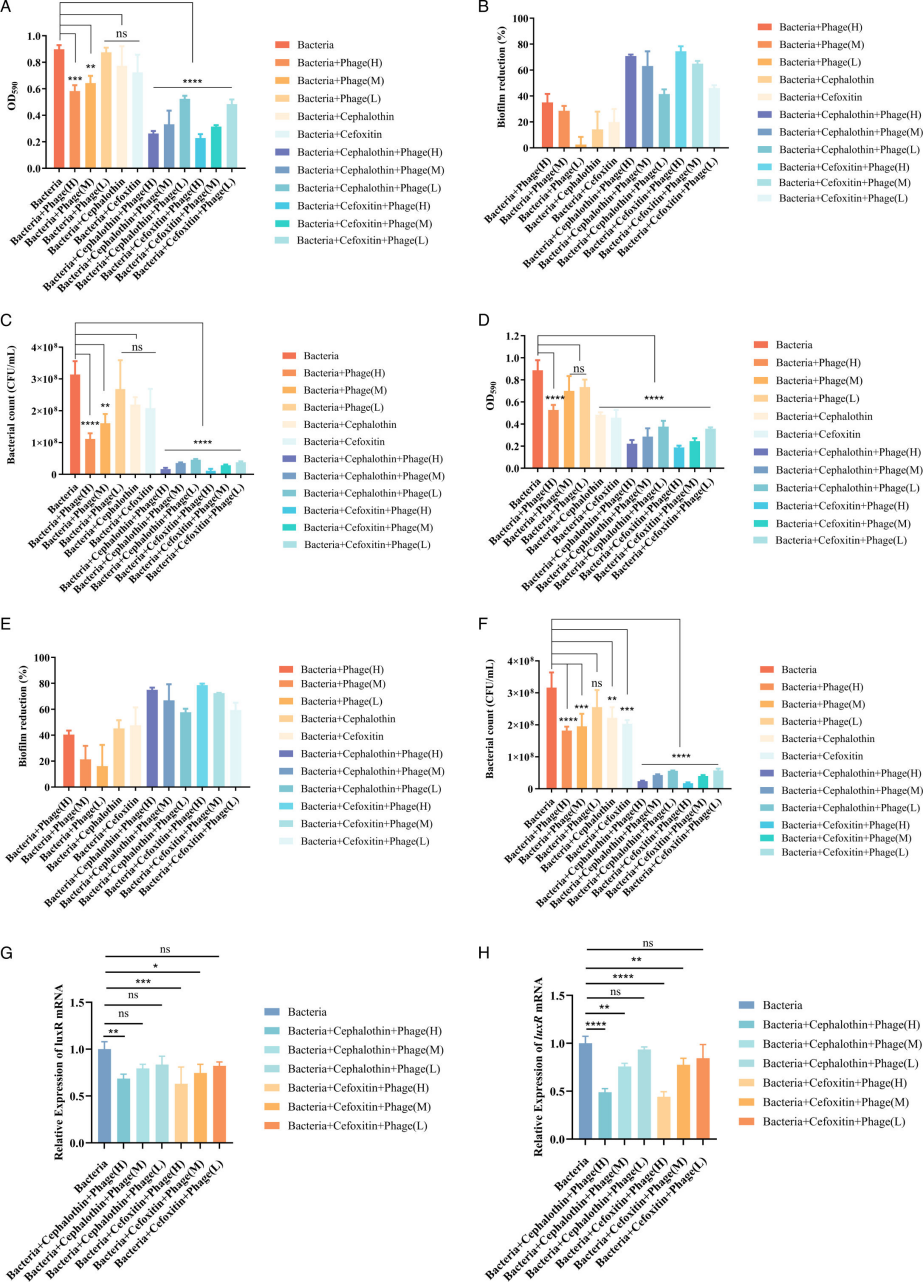
Effect of combined treatment with phage vB_C4 and antibiotics on mature biofilm and biofilm formation of *A. veronii* C4. (**A**) Treatment of mature biofilm of *A. veronii* C4 with phage vB_C4 alone or in combination with antibiotics. (**B**) Destructive effect on mature biofilm. (**C**) Viable count of *A. veronii* C4 bacteria after treatment of mature biofilm with phage vB_C4 and antibiotics. (**D**) Biofilm formation of *A. veronii* C4 under treatment with phage vB_C4 alone or in combination with antibiotics. (**E**) Inhibitory effect on biofilm formation. (**F**) Viable count of *A. veronii* C4 bacteria in the biofilm after 48 h of treatment with phage vB_C4 and antibiotics. (**G**) Changes in *luxR* transcription levels during the maturation of biofilms. (**H**) Changes in *luxR* transcription levels during biofilm formation. The antibiotic concentration used was 0.5 × MIC of cephalothin or 0.5 × MIC of cefoxitin. Phage doses were categorized into three levels: low dose (MOI = 0.01), medium dose (MOI = 1), and high dose (MOI = 100), denoted by L, M, and H, respectively. Data are represented as the mean of three biological replicates, with error bars representing the standard deviation (SD) of the mean. * indicates *P* < 0.05, ** indicates *P* < 0.01, *** indicates *P* < 0.001, **** indicates *P* < 0.0001, and NS indicates no significant difference.

Although low doses (MOI = 0.01) of vB_C4 and sub-inhibitory concentrations of antibiotics alone had no significant effect on the removal of mature biofilms (Fig. 6A and B), medium doses (MOI = 1) and high doses (MOI = 100) of phage vB_C4 alone partially and effectively eradicated mature biofilms. This resulted in a reduction of biofilm volume by 28.59% and 35.02%, respectively (Fig. 6B), and achieved more than a twofold reduction in bacterial counts (Fig. 6C). In contrast, the combination of different doses of phage vB_C4 and antibiotics produced a synergistic effect that significantly eradicated established biofilms, achieving up to a 74.58% reduction in biofilm volume and approximately a 28-fold reduction in bacterial counts (Fig. 6C).

According to Fig. 6D through F, phage vB_C4 at low doses (MOI = 0.01) and medium doses (MOI = 1) alone did not prevent biofilm formation. However, antibiotics alone significantly inhibited the growth of *A. veronii* C4, leading to a substantial inhibition of biofilm formation. The combination of phage vB_C4 and antibiotics effectively prevented biofilm formation and produced better inhibition. The same results were obtained using the dilution plate counting method (Fig. 6E and F). Studies have shown that the *luxR* gene is involved in the process of biofilm formation (24). We tested whether the combination treatment affected *luxR* gene expression and thus inhibited bacterial biofilms. The results indicated that the level of *luxR* gene transcription was significantly reduced at high doses (MOI = 100) of phage vB_C4 in combination (Fig. 6G and H).

## DISCUSSION

The misuse of antibiotics has led to an increasing prevalence of multidrug-resistant bacteria. The development of new broad-spectrum antibiotics is infrequent due to the high costs associated with their creation. Even when new antibiotics are developed, pathogenic bacteria eventually develop resistance to them. This spread of drug resistance among bacterial pathogens poses serious challenges in clinical settings, resulting in significant mortality and morbidity. Consequently, there is a renewed interest in utilizing the antimicrobial properties of phages as therapeutic tools.

*A. veronii* C4, a significant pathogen, has developed continuous drug resistance due to the irrational use of various antibiotics in its treatment, complicating disease prevention and control. In response to this issue, this study targeted *A. veronii* C4 and isolated a specific phage from hospital wastewater, named vB_C4. In addition to encoding proteins essential for phage survival, vB_C4 also encodes hydrolases that digest bacterial cell wall polysaccharides, a phage lytic protein (endolysin) that degrades extracellular polysaccharide (EPS), and GumC, a protein associated with cell wall lysis (Fig. 1). Studies have shown that phage-encoded EPS-degrading enzymes enhance phage infections and bolster anti-biofilm and antimicrobial effects. These enzymes have potential roles in preventing and treating bacterial biofilm-associated infections, and several phage endolysins have entered clinical trials. Genome-wide BLAST comparisons revealed that phage vB_C4 shares more than 97% gene identity and over 87% query cover with *Aeromonas* phage phiA034 (OP792756.2) and others. Comparative genomic analysis was conducted on vB_C4 and six selected phages (Fig. 2). The phylogenetic tree demonstrated high values of self-expansion in each branch, indicating high credibility. The genome of *Aeromonas* phage vB AhyS-A18P4 was markedly different from other phages, diverging earliest. vB_C4 appeared in the second clade and shared a recent common ancestor with the third clade phages (BUCT551, pAEv1812, and phiA034), suggesting they may share similar traits (Fig. 2B).

*In vitro* antibacterial experiments revealed that medium and high doses of phage vB_C4 (MOI ≥ 1) effectively reduced the concentration of its host bacterium, demonstrating significant antibacterial effects (Fig. 3D). However, low doses (MOI ≤ 0.1) showed antibacterial activity only during the initial incubation period (Fig. 3E). Phage vB_C4 did not completely eliminate *A. veronii* C4 but significantly reduced its population density. This limitation arises because phages are self-replicating viruses that rely on the host bacterium’s metabolic system to produce progeny phages. Once outside the host bacteria, phages neither grow nor replicate, leading to their rapid decline (25).

Combination therapy presents a more effective approach to controlling and killing bacteria while reducing the evolution and spread of resistance compared to using phages or antibiotics alone (26). In recent years, phage-antibiotic combination therapy has garnered widespread attention. Phages can destroy the defenses of bacteria, for example, phages use depolymerase to degrade the biofilm matrices, thereby promoting the diffusion and penetration of antibiotics, and allowing low doses of antibiotics to potentially eliminate bacteria (27). The presence of phages enhances the efficacy of the three main classes of antibiotics, namely quinolone, β-lactams, and tetracycline antibiotics (28). Phage PAM2H also increases the sensitivity of *Pseudomonas aeruginosa* to ciprofloxacin or gentamicin (29), thereby enhancing the therapeutic effect. Cephalothin (a first-generation cephalosporin) and cefoxitin (a second-generation cephalosporin) were selected in combination with different doses of phages to evaluate their antibacterial effects on *A. veronii* C4. The combination of phage and cephalothin/cefoxitin significantly reduced bacterial turbidity, with higher doses exhibiting more pronounced inhibitory effects (Fig. 4C and 5A). Viable bacterial counts further indicated a significant reduction in the number of *A. veronii* C4 (Fig. 4). However, the antibacterial effect of cephalothin combined with high-dose phage (MOI = 100) gradually diminished over time, and the inhibitory effect at 24 h was not significantly different from that of the low-dose combination (MOI = 0.01) (Fig. 4C). There may be a risk of phage resistance during long-term use of phage therapy. Researchers have employed the following strategies to reduce the incidence of phage resistance: (i) phage cocktail therapy (30). For example, in the prevention and control of *Salmonella enteritidis*, a cocktail of four phages that recognize different receptors can significantly delay the emergence of phage-resistant strains (31). (ii) Phage alternation therapy (32). In addition, bacteriophage therapy is often used as an adjuvant therapy, so the emergence of resistance may be manageable (33).

In natural environments, pathogenic bacteria rarely exist as planktonic cells in liquids but are usually encased in a polysaccharide matrix known as a biofilm, forming colonies on surfaces or semi-solids (34). Biofilm formation allows bacteria to survive in extreme environments, including exposure to antibiotics, leading to increased resistance and reduced effectiveness of antibiotic treatments (34, 35). Combining phage and antibiotic treatments is effective in preventing and controlling biofilm formation (36). In this study, we evaluated the effects of different doses of phage combined with cell wall-targeting antibiotics on the eradication of mature biofilms and the prevention of biofilm formation in *A. veronii* C4. As shown in Fig. 6, the combination of phage vB_C4 with cephalothin or cefoxitin effectively removed mature biofilms and prevented their formation, reducing both the bacterial density within the biofilm and the biofilm biomass, thereby demonstrating significant anti-biofilm activity.

In summary, the combination of phage and antibiotics may be an effective strategy for controlling clinical infections caused by biofilm-forming bacteria. Our research provides a foundation for evaluating the parameters necessary for using phage-antibiotic combination therapy to treat biofilm-associated infections. Future research should focus on the practical application of different phage and antibiotic combinations for the treatment of biofilm infections.

## MATERIALS AND METHODS

### Bacteria and culture conditions

*A. veronii* C4 was isolated and preserved in our laboratory (37). A single colony of *A. veronii* on LB agar plates was inoculated into 5 mL of fresh LB medium and incubated with shaking for approximately 12 h at 30°C and 150 rpm. The bacterial suspension was centrifuged to remove the supernatant and resuspended in fresh LB medium. Bacterial colony-forming units (CFU) were determined using the plate dilution method (38), and the serial dilution of bacteria was used for subsequent experiments.

### Phage enrichment and purification

The phage vB_C4, which specifically targets *A. veronii* C4, was isolated from hospital wastewater samples collected in Haikou City, Hainan Province, China. The upper agar gel block containing phage spots was selected and resuspended in SM buffer (50 mM Tris-HCl, pH 7.5, 150 mM NaCl, 10 mM MgCl_2_, and 2 mM CaCl_2_), incubated in a shaker for 4 h, and then centrifuged at 12,000 rpm for 5 min. The resulting supernatant was serially diluted and incubated with the host bacteria, and the double-layer plate method was used to obtain single phage spots (39). High-titer phage lysates were obtained by infecting log-phase *A. veronii* with purified phage (40). The phage lysate was centrifuged, and the supernatant was filtered through a 0.22 µm filter membrane and stored at 4°C for later use.

### Genomic sequencing and genome mapping

Phage DNA was extracted using the E.Z.N.A. Viral DNA Kit (OMEGA) (Omega Bio-Tek, Guangzhou, China). Genome sequencing was performed on an Illumina NovaSeq 6000 genome sequencer. Quality control of the raw data were conducted using Trimmomatic v0.36. Genome assembly optimization was performed using ABySS v2.2.0 with multiple Kmer parameters. GapCloser v1.12 was employed to fill gaps within the genome and correct single-base polymorphisms for the final assembly. Coding genes were predicted using GeneMarkS software and annotated based on comparisons with the NR and eggNOG databases. Non-coding RNA (ncRNA) prediction was conducted using Infernal v1.1.14 software. On the website https://card.mcmaster.ca/ check the genes encoding antibiotic resistance in the phage genome sequence. Genome circular mapping was implemented using Proksee.

### Comparative genome analysis and phylogenetic tree construction of phage vB_C4

The phage genome was subjected to a BLAST search in NCBI’s nr database. Six phages with the highest scores from different lineages were selected, and their genome information and protein sequences were downloaded. The phage protein sequences were inputted into Orthofinder v2.5.2 to screen the homologous genomes and obtain core, auxiliary, and specific genes. The core and auxiliary genes of the phages were clustered using the “complete” method to determine the similarity among different phages, and the comparative genome results were visualized using Chiplot.

Multiple sequence alignment of phage core single-copy genes was performed using MAFFT v.7.508, and the results were trimmed using Trimal v1.4.rev15. The trimmed sequences were concatenated in tandem to generate a multi-gene concatenation result. These concatenated sequences were inputted into IQ-TREE v2.1.4-beta for phylogenetic tree construction, which was further refined with 1,000 bootstrap replicates. The phylogenetic tree was visualized using iTOL.

### Host range determination of phage vB_C4

The laboratory-preserved pathogens (*S. aureus*, *Salmonella* S504, *S. agalactiae*, *V. alginolyticus*, *P. aeruginosa*, and *A. veronii*) were cultured to the logarithmic growth stage. A volume of 100 µL of the bacterial solution was spread on an LB agar plate and left for 15 min to allow the solution to dry. Subsequently, 5 µL of phage stock solution at a concentration of 10^10^ PFU/mL was added dropwise to the center of the plate. The plate was then incubated at 37°C overnight, and phage spots were observed the following day.

### Stability determination of phage vB_C4 in UV, chloroform, and detergent

To investigate the sensitivity of vB-C4 to ultraviolet light, 10 mL of a phage suspension with a concentration of 10^10^ PFU/mL was poured into a sterile culture dish and exposed to ultraviolet radiation. Samples were taken every 10 min for a total duration of 60 min. After sample filtration, the phage potency was determined using the double-layer plate method.

In order to study the effect of organic solvents on vB_C4, chloroform at a final concentration of 5%, 0.1% SDS, and 0.3% TritonX-100 were added to the phage stock solution at a concentration of 10^10^ PFU/mL. The control group received an equal amount of phosphate-buffered saline (PBS) and was left to stand for 1 h at room temperature. The mixture was then centrifuged for 5 min at 10,000 × *g*, and the supernatant was collected for potency measurement using the double-layer plate method.

### Safety assessment of phage vB-C4 on CaCO_2_ intestinal epithelial cells

A total of 10^5^ CaCO_2_ cells were inoculated into each well, with 100 µL of culture medium per well. The cells were cultured at 37°C for 12 h until they adhered to the well walls. The original culture medium was removed, and medium containing different concentrations of phage vB_C4 was added. Fresh culture medium without phage was added to the control group, which was incubated for 24 h. Subsequently, the cells were washed more than three times with PBS, and cell survival rates were determined using the CCK-8 kit. Three replicates were set up for each group.

### Determination of MIC of antibiotics and the effect of antibiotics on phage proliferation

The MIC of antibiotics against *A. veronii* C4 was determined using the microbroth dilution method. The effect of antibiotics on phage proliferation was studied using the double-layer culture method. Phage vB_C4 (~10⁶ PFU/mL) was mixed with host *A. veronii* C4 (~10⁶ CFU/mL) and incubated at 30°C for 24 h with or without the addition of antibiotics. Phage spot counting was then performed.

### *In vitro* antibacterial activity assay of phage vB_C4

A total of 100 µL of *A. veronii* C4, at a concentration of 10⁶ CFU/mL, was added to each well of a 96-well plate. Phages at varying concentrations were added to the corresponding wells, with three replicates performed for each experimental group. In the control group, equal volumes of bacterial solution and PBS were added. The optical density at 600 nm (OD_600_) was measured using a microplate reader.

### *In vitro* antibacterial activity of different doses of phage vB_C4 in combination with antibiotics

A total of 100 µL of *A. veronii* C4 at a concentration of 10⁶ CFU/mL was added to each well of a 96-well plate. Equal volumes of antibiotics (0.5 × MIC cephalothin or cefoxitin), phage solutions at three different MOI levels, and combinations of phage and antibiotic were added to their respective wells. In the control group, an equal volume of PBS was added to the bacterial solution. The number of viable bacteria after 20 h of culture was determined using the agar dilution plate counting method.

### Evaluation of the effect of *in vivo* combination therapy in mice

Male mice (4–6 weeks old) were randomly divided into five groups, of which four groups were injected intraperitoneally with *A. veronii* C4 at a concentration of 10^6^ CFU/mL. The control group was injected intraperitoneally with an equal volume of sterile PBS. After 4 h of inoculation, 100 µL of phage vB_C4 (10^8^ PFU/mL), 0.5 × MIC cefoxitin, or a mixture of vB_C4 (10^8^ PFU/mL) and 0.5 × MIC cefoxitin were administered intraperitoneally. Mice in the control group were intraperitoneally injected with the same volume of sterile PBS. The mice were executed 24 h later, and the heart, liver, spleen, lung, and kidney were weighed and divided into two portions. One portion was fixed with 4% paraformaldehyde for paraffin sectioning and hematoxylin and eosin (H&E) staining, while the other portion was ground and coated on agar plates for counting.

### Effects of phage vB_C4 and antibiotics on biofilm formation and mature biofilms of *A. veronii* C4

**Fig 7:**
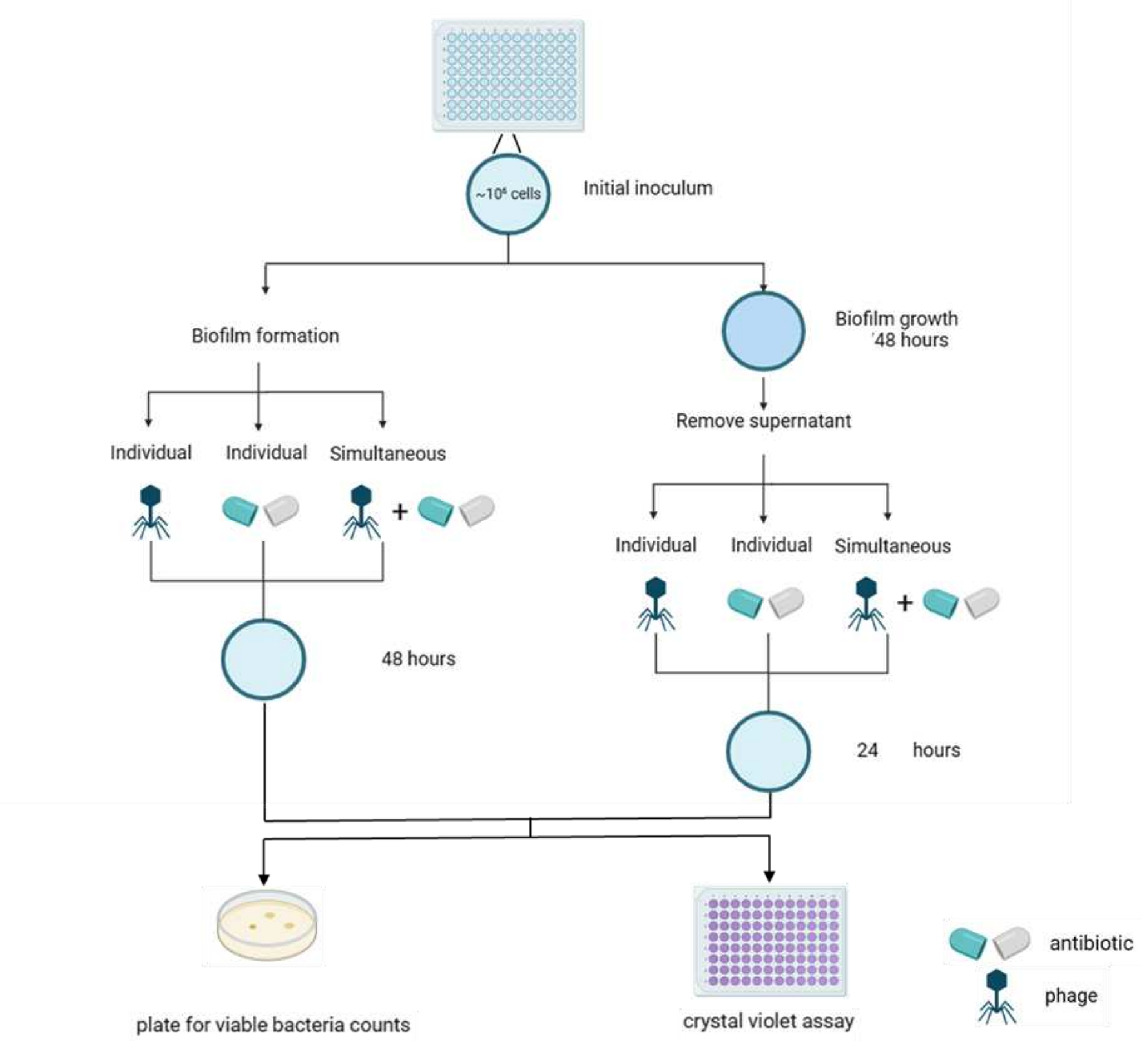
Schematic diagram illustrating the experimental setup to assess the effects of various doses of phages and antibiotics (cephalothin or cefoxitin) on the biofilm of *A. veronii* C4.

To evaluate the effects of phage vB_C4 in combination with antibiotics (cephalothin or cefoxitin) on biofilm formation, the procedure is illustrated in Fig. 7. Briefly, a 100 µL suspension of *A. veronii* C4 at a concentration of 10⁷ CFU/mL was inoculated into 96-well plates. The experimental groups were added with antibiotics, phage, or a combination of both, and were then incubated at 30°C for 48 h. The control group received an equal volume of PBS. Total biomass was quantified using the crystal violet staining method. Additionally, the number of viable bacteria within the biofilm was determined using the plate counting method.

The impact of phage vB_C4 and antibiotic combinations on the reduction of mature biofilms was assessed using established methods. *A. veronii* C4, at a concentration of 10⁷ CFU/mL, was first incubated at 30°C for 48 h to form mature biofilms. The supernatant was aspirated to remove planktonic bacteria, and the biofilm was washed two times with PBS. The mature biofilms were then treated with antibiotics, phage, or a combination of phages and antibiotics, and incubated at 30°C for 24 h under static conditions. The content of the biofilm and the number of viable bacteria were measured under these different conditions.

### Real-time fluorescence quantitative PCR

Total RNA was extracted using the Bacterial Total Ribonucleic Acid Extraction Kit (Sangon Biotech, Shanghai, China). Residual genomic DNA was removed with DNase I, and cDNA was synthesized by reverse transcription using HiScrip II QRT SuperMix (Vazyme, Nanjing, China). Add cDNA, ChamQ SYBR color qPCR Master mix (Vazyme, Nanjing, China), and primers for *luxR* amplification (F: 5′-ATCTTCTGGAACAAGCTCG-3′; R: 5′-CAAACATCAGATCGGAGT-3′). The housekeeping gene *gyrB* was used as a reference for data normalization. Quantitative real-time polymerase chain reaction (qRT-PCR) assays were performed using a LightCycler96 (Roche, Basel, Switzerland). Finally, relative expression of mRNAs was calculated using the comparative cycle threshold (CT) method.

### Statistical analysis

The presented data were obtained from a minimum of three independent experiments and were expressed as means ± standard deviation. The statistical analysis method was the one-way analysis of variance test, statistical analysis of results was shown with GraphPad Prism version 8.3 (GraphPad Software Inc., San Diego, CA. USA), using a *P* value of <0.05 as statistically significant.

## Data Availability

The data for bacteriophage vB_C4 have been released on NCBI, GenBank under PQ676986.1.
